# Effect of intra-peritoneal induction of ascites fluid on the rate of postoperative intraabdominal adhesion in a rat model

**DOI:** 10.1016/j.amsu.2022.104129

**Published:** 2022-07-11

**Authors:** Zahra Amirian, Mahmoud Zardast, Mohsen Najmodini, Mohammadreza Ghasemian Moghadam

**Affiliations:** a Surgical Resident, School of Medicine, Birjand University of Medical Sciences, Birjand, Iran; b Medical Toxicology & Drug Abuse Research Center, Birjand University of Medical Sciences, Birjand, Iran; c Cardiovascular Diseases Research Center, Department of Surgery, School of Medicine, Birjand University of Medical Sciences, Birjand, Iran; d Clinical Research Development Unit, Imam Reza Hospital, Birjand University of Medical Sciences, Birjand, Iran

**Keywords:** Intra-abdominal adhesions (IAA), Low SAAG, High SAAG, Ascites fluid

## Abstract

**Introduction:**

Intra-abdominal adhesions (IAAs) are secondary to peritoneal injuries such as previous surgery or intra-abdominal infections (IAIs). Accordingly, it is crucial to employ fitting techniques to minimize the likelihood of adhesions in any surgery. Due to a paucity of similar data available, this study sought to explore the effects of induced high serum ascites albumin gradient (SAAG) and low serum ascites albumin gradient (SAAG) on the rate of post-operative microscopic and macroscopic adhesion in a mouse model.

**Material and methods:**

Sixty mice were compared in six groups of ten each. Control groups (1 &4) received normal saline, groups 2&5 received high SAAG ascites fluid, and groups 3&6 received low SAAG ascites fluid intraperitoneally. These groups underwent exploratory laparotomy on day zero, followed by the same procedure on the 10th (groups 1,2,3) and the 30th (Groups 4,5,6) day of surgery. Then, microscopic and macroscopic IAAs were evaluated. Data were analyzed in SPSS software and compared with a p-value less than 0.05.

**Results:**

By comparison, the least microscopic and macroscopic IAAs after 10 and 30 days were found in the low SAAG ascites group. Revealing a statistically significant difference compared to the other two groups (P = 0.01). After 10 days of surgery, macroscopic IAA in the high SAAG group was significantly lower compared to the control and Low SAAG ascites groups.

**Conclusion:**

Intraabdominal low SAAG ascites fluid can significantly decrease the probability of postoperative fibrosis and adhesion band formation.

**Protocol number:**

IR. BUMS.REC.1399.503.

## Highlights

• Regarding macroscopic adhesions 10 days and 30 days post laparotomy, low SAAG ascites had significantly prevented IAAs.
• The least microscopic and macroscopic IAAs, 10 and 30 days after surgery, were found in the low SAAG ascites group.
• The results of reduced IAAs using low SAAG ascites can be beneficial to the primary prevention of postoperative adhesions.

## 1 Introduction

Peritoneal adhesions are bands of fibrous tissue that form among naturally detached organs, mainly in the abdominal cavity [1]. Small bowel obstruction (SBO) is largely brought on by intra-abdominal adhesions (IAAs), which account for 65 to 75% of cases, mostly in the ileum. Lower abdominal surgery has a higher risk of causing adhesions. Postoperative adhesions have the potential to cause severe side effects, including intestinal obstruction (IO), female infertility, pelvic and abdominal discomfort, and secondary effects such protracted and risky subsequent abdominal surgery [2]. Research has revealed that the rate of adhesions after abdominal surgery is 51%, 66%, and 22% after obstetric and gynecological surgery, gastrointestinal surgery, and urological surgery, respectively [3]. Urinary incontinence, persistent abdominal pain, and problems conducting reoperations are some other consequences.

In Iran, according to studies, adhesive bands represent the major cause of SBOs. Many patients suffer from recurrent acute SBO (ASBO), culminating in recurrent laparotomies [4]. Adhesion further accounts for 15–20% of infertility in women and can inflict diminished mobility and physical occlusion of the fallopian tubes. Once again, it may prevent the mobility of eggs and raise the possibility of an ectopic pregnancy. Additionally, a significant factor in the failure of four-segment fallopian tube repair surgery is postoperative adhesion [5]. In general, after rectal surgery, left hemicolectomy (LH), and/or total colectomy (TC), 11% of SBO episodes occur within the first year. After 10 years, however, SBO episodes account for up to 3% of all cases in laparotomy surgeries. When the peritoneal surface is damaged due to surgery, thermal shock, ischemia, inflammation, or a response to a foreign substance, a peritoneal adhesion develops. Additionally, within 10 years after the disease's beginning, ascites appears in more than 60% of individuals with compensated cirrhosis (CC). Differential diagnosis is essential for improved cirrhosis therapy. Analysis of peritoneal fluid (also known as ascitic fluid) is also crucial [6]. Ascites occurs when excessive fluid builds up in the peritoneal cavity. In general, the peritoneal cavity retains a small volume of high-protein-content fluid (<50 ml). Fluid accumulation in the peritoneal cavity and thus ascites follows different mechanisms. A total of 85% of ascites are caused by portal hypertension associated with cirrhosis. Similarly, 15% of ascites are in terms of non-cirrhotic intra-abdominal conditions such as malignancy, infection, and heart and kidney failure. Based on ascitic fluid total protein, transudative ascites cannot accurately pinpoint the causes. In fact, little is understood regarding the physiological formation of ascites fluid. In response to it, SAAG is a new approach to classify ascites into two categories: 1) high SAAG (>1.1 g/dL) in portal hypertension cases, and 2) low SAAG (<1.1 g/dL) in ascites cases, irrelevant to portal hypertension.

SAAG determines the oncotic pressure induced by serum albumin on ascites fluid albumin, which is equivalent to the high hydrostatic pressure gradient between the portal system and ascites fluid [7].

Unprecedentedly, this study aims to explore whether the presence of IAA can prevent post-operative adhesions caused by peritoneal injury [8].

Additionally, the primary line of defense against adhesions has always been precise surgical techniques. Adhesions are less likely to form when there is precise and regulated homeostasis, less tissue injury and irrigation, less heat damage, and regular washing. To prevent direct contact between the intestines and the abdominal wall, it is best to place an omentum over the intestines before closing the laparotomy incision [9]. There are two strategies to prevent or reduce adhesions. One strategy is to minimize surgical injuries. Reducing excessive use of catheters, lasers, and retractors, as well as mild handling of tissues and preventing tissue rupture or ischemia are helpful. Typically, less tissue damage is associated with less likely to induce adhesions in laparoscopic surgery [1]. Besides, there are only three FDA-approved products to reduce adhesion.

It is still difficult to completely avoid adhesions even using precise techniques and processes. IAAs and pelvic adhesions brought on by surgery may result in severe pain, IO, decreased fertility, and challenges with reoperations. Therefore, it is essential to follow the right measures to reduce the possibility of adhesions after any operation. There are uncertain and contradictory results in the literature review of the animal and human trials carried out utilizing various techniques and materials to reduce adhesion. Indeed, preventing postoperative adhesions is nonetheless an art than a science. Concerning the paucity of similar data available, this study aims to scrutinize the effects of induced high SAAG and low SAAG on the rate of postoperative microscopic and macroscopic adhesion in a mouse model.

## 2 Methods

• In this experimental randomized controlled animal trial, Sixty Syrian male rat weightings 40±5gr were allocated to six groups of 10 rats each through random allocation rule. It considers the ethical guidelines of the animal ethics committee of Birjand University of Medical Sciences (Ref: IR. BUMS.REC.1399.503). Sample size was calculated using the sample size formula for comparison of three means, at α = 0.05 and B (power) = 80%. Rats included in the study were all Syrian males. The rats were excluded if they died or were unwell (observation of disease signs and symptoms, such as increased porphyrin, coryza symptoms, tachypnea, anorexia, nodules or papules anyplace on the body, alopecia, agitation, and …); these criteria were predetermined in advance. The surgeon who performed the first operation was not the same person who assessed the laparotomy. All surgical procedures were conducted in sterile condition and the evaluation on relaparotomy was completed by a different operator. The same conditions were established on the 10th and 30th day of surgery. We scrubbed alcohol on the rat's abdomen, then injected 0.2 cc of ketamine and 0.1 cc of xylazine into the peritoneum. After a brief period of anesthesia, we did PREP & DREP on the abdomen. We then used a 10-gauge razor blade to make a 2 cm midline incision in the belly, which caused the peritoneum to open. In all instances, the peritoneum received many sharp abrasions in the identical ways. The abdominal wall was repaired with nylon thread 0-5 using the suture locking method after three cc of the necessary fluid was injected into the peritoneum using a syringe. Then, the rats were kept in their standard plastic cages (2 rats per cage, 50✕50✕40 cm in size) under controlled temperature (21 ± 2 °C), 58–65% humidity and a 12/12 light/dark cycles with food and water accessible. Every three days 2 cc of the fluid of each group was charged by reinjection into the peritoneum with a syringe. (Based on the pilot study findings on intraperitoneal fluid loss rate). 10 and 30 days after initial surgery the rats were killed and underwent another laparotomy in groups 1, 2, 3 and 4, 5, 6, respectively, to assess macroscopic and microscopic adhesions, which were scored by a surgery resident who had no knowledge of the animals' group assignment.

### 2.1 Study groups

Group I: Positive control mice. 3 ml of normal saline was poured into the peritoneal cavity and the abdominal wall was then repaired.

Group II: Before repairing the abdominal wall, mice received 3 ml of high SAAG human ascites fluid poured into their peritoneal cavity.

Group III: Before repairing the abdominal wall, mice received 3 ml of low SAAG human ascites fluid poured into their peritoneal cavity.

Mice in groups I to III underwent laparotomy again after housing them in the same condition for 10 days, and for them, IAA was observed via macroscopic and microscopic adhesion scoring systems.

Group IV: Positive control mice. 3 ml of normal saline was poured into the peritoneal cavity and the abdominal wall was then repaired.

Group V: Before repairing the abdominal wall, mice received 3 ml of high SAAG human ascites fluid poured into their peritoneal cavity.

Group VI: Before repairing the abdominal wall, mice received 3 ml of low SAAG human ascites fluid poured into their peritoneal cavity.

Every three days 2 cc of the fluid of each group was charged by reinjection into the peritoneum with a syringe. (Based on the pilot study findings on intraperitoneal fluid loss rate).

Mice in groups IV to VI underwent laparotomy again after keeping them in the same condition for one month, and for them, IAA was assessed via macroscopic and microscopic adhesion scoring systems.

Samples were taken with a camera and scored using the adhesion scoring method developed by Aksoy et al. (Table 1) [7] for the macroscopic section. We utilized the grading method from Lashkarzadeh et al. work to determine the adhesion grade microscopically (Table 2). [8]The analysis of two types of ascites fluid used, is presented in Tables 3 and 4.

**Table 1:** Adhesion scoring system according to the study of Aksoy et al. Published in the journal European surgical research.

Degree of adhesion	Type of adhesion band
0	No adhesion
1	Thin adhesive that can be easily removed
2	Thin adhesive bands that are gathered in one area
3	Thin adhesive bands that are widely distributed in the abdomen
4	Adhesion of organs to the abdominal wall with abundant adhesive bands

**Table 2:** Pathology grading used in the study of Lashkarzadeh et al.

Pathology grading	category
1	Inflammatory markers including: PMN infiltration clot formation and macrophage Lack of collagen accumulation and new angiogenesis (acute phase of inflammation)
2	Replication markers include: fibroblast infiltration, collagen and progoglycan synthesis, new angiogenesis and pmn reduction, and chronic granulation tissue production of the inflammatory phase)
3	Maturity markers include: reduction of cells and arteries and scar production

**Table 3:** Analysis of the high SAAG ascitic fluid.

Parameter	Level	Parameter	Level
Calcium (Ca)	6.7	Sodium (Na)	134
Blood sugar (BS)	77	Lipase	32
Amylase	29	Potassium (K)	4
Lactate dehydrogenase (LDH)	127	PH	8.0
Albumin	1.1	Protein	2.4
Blood Urea Nitrogen (BUN)	38	Creatinine (Cr)	1
White Blood Cell (WBC)	20	Red Blood Cell (RBC)	2–3

**Table 4:** Analysis of the Low SAAG ascitic fluid.

Parameter	Level	Parameter	Level
Calcium (Ca)	8	Sodium (Na)	137
Blood sugar (BS)	64	Lipase	53
Amylase	56	Potassium (K)	3.4
Lactate dehydrogenase (LDH)	1803	PH	8.1
Albumin	2.3	Protein	3.4
Blood Urea Nitrogen (BUN)	58	Creatinine (Cr)	1.6
White Blood Cell (WBC)	800	Red Blood Cell (RBC)	4–5

There were no exclusions in animals and data. The work was reported in accordance with ARRIVE guidelines (Animals in Research: Reporting in Vivo Experiments) and Standards of Reporting Trials (CONSORT) Guidelines (9).

Using SPSS version 24, descriptive statistics and analytical tests were used to evaluate the data. The Kolmogorov-Smirnov test was used to determine if the data were normal, and the Kruskal Wallis and Bonferroni-corrected Mann-Whitney U tests were used to analyze the data. P-values under 0.05 were regarded as significant.

## 3 Results

According to Table 5, there is no significant difference in the mean weight of the mice between groups. The least percentage of microscopic IAA in samples obtained 10 days after surgery is observed in the low SAAG group, with a significant difference with other groups (P < 0.001). After 30 days, the least microscopic IAA is observed in the low SAAG group (P < 0.001).

**Table 5:** Comparison of the mean weight and microscopic IAA, 10 and 30 days after surgery in groups under study.

Variable		Control (n = 20)	High SAAG (n = 20)	Low SAAG (n = 20)	p-value
Weight (gr)		3.43 ± 31.10	3.47 ± 31.10	3.56 ± 30.75	0.92
Microscopic IAA (%)	10 days	13.76 ± 18.3	5.29 ± 18.3	7.3 ± 9.14	<0.001
	30 days	14.16 ± 83.3	9.62 ± 25	8.03 ± 11.63	<0.001

The least percentage of macroscopic IAA in samples obtained 10 days after surgery is observed in the low SAAG ascites fluid, with a significant difference with other groups (P = 0.01). (Table 6).

**Table 6:** Comparison of the macroscopic IAA grading, 10 days after surgery in groups under study.

Macroscopic IAA/Group	Control (n = 20)	High SAAG (n = 20)	Low SAAG (n = 20)	p-value
Grade 0	0 (0)	1 (20%)	4 (80%)	0.01
Grade I	3 (21.4%)	8 (57.1%)	3 (21.4%)	
Grade II	4 (50%)	1 (12.5%)	3 (37.5%)	
Grade IV	2 (100%)	0 (0)	0 (0)	
Grade V	1 (100%)	0 (0)	0 (0)	
Mean rank	21.85	12.65	12	

According to Table 7, the least percentage of macroscopic IAA in samples obtained 30 days after surgery is observed in the low SAAG and high SAAG groups, respectively, (P = 0.02).

**Table 7:** Comparison of the macroscopic IAA grading, 30 days after surgery in groups under study.

Macroscopic IAA/Group	Control (n = 20)	High SAAG (n = 20)	Low SAAG (n = 20)	p-value
Grade 0	0 (0)	1 (33.3%)	2 (66.7%)	0.02
Grade I	2 (28.6%)	2 (28.6%)	3 (42.9%)	
Grade II	2 (20%)	3 (30%)	5 (50%)	
Grade III	1 (25%)	3 (75%)	0 (0)	
Grade IV	3 (75%)	1 (25%)	0 (0)	
Grade V	2 (100%)	0 (0)	0 (0)	
Mean rank	20.6	15.56	10.25	

According to Spearman correlation test (Table 8), the nexus among the days after surgery and the rate of microscopic and macroscopic IAA is significant specifically in the high SAAG group (P = 0.01).

**Table 8:** The relationship between days after surgery and the rate of microscopic and macroscopic IAA in groups under study on 10th and 30th days.

Group	Macroscopic		Microscopic	
	Test statistics	p-value	Test statistics	p-value
Control (days 10 and 30)	0.23	0.32	0.43	0.06
High SAAG (days 10 and 30)	0.54	0.01	0.41	0.07
Low SAAG (days 10 and 30)	0.24	0.3	0.19	0.41

**Fig. 1:**
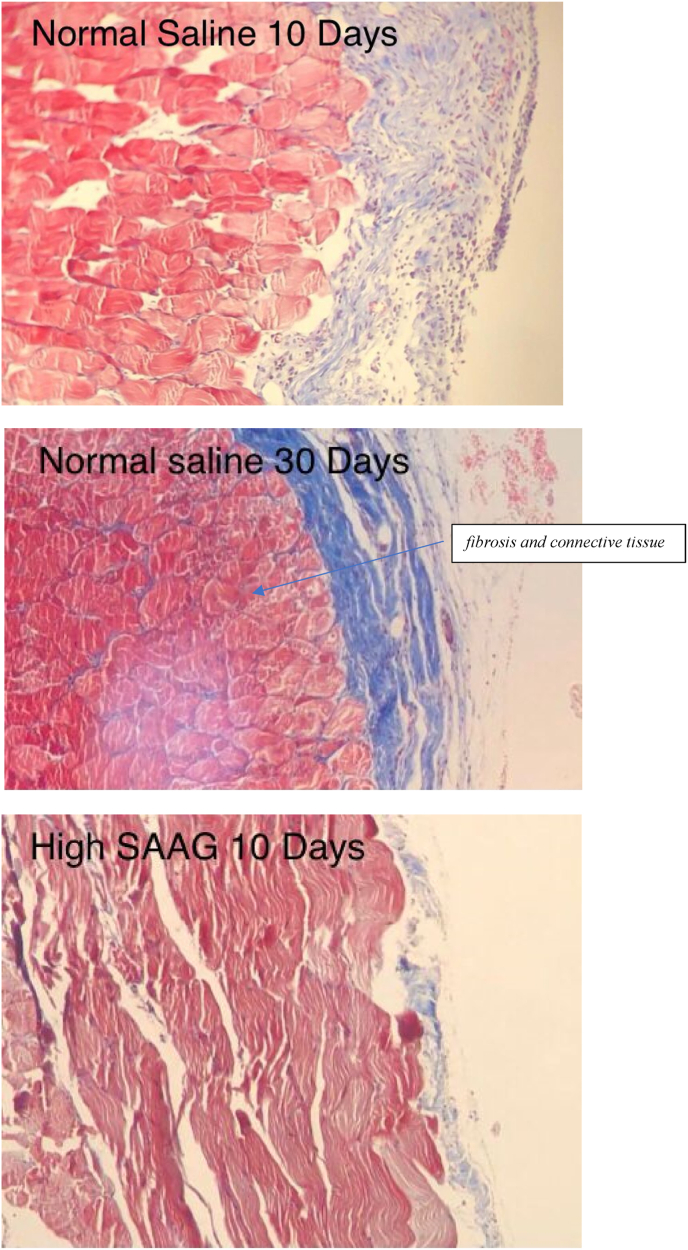

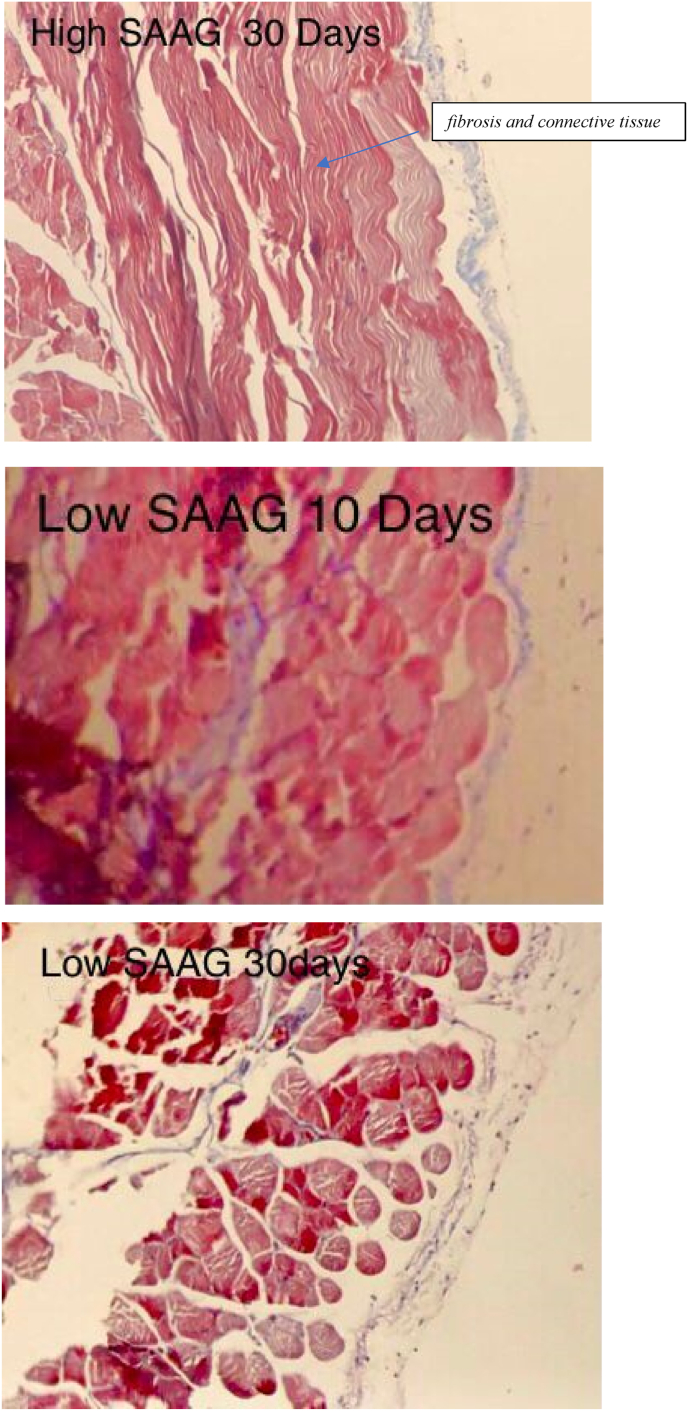
**Masson's trichrome** stain. Specifically, to distinguish cells from surrounding connective tissue. Connective tissue is stained blue, nuclei are stained dark red/purple, and cytoplasm is stained red/pink. (For interpretation of the references to colour in this figure legend, the reader is referred to the Web version of this article.)

**Fig. 2:**
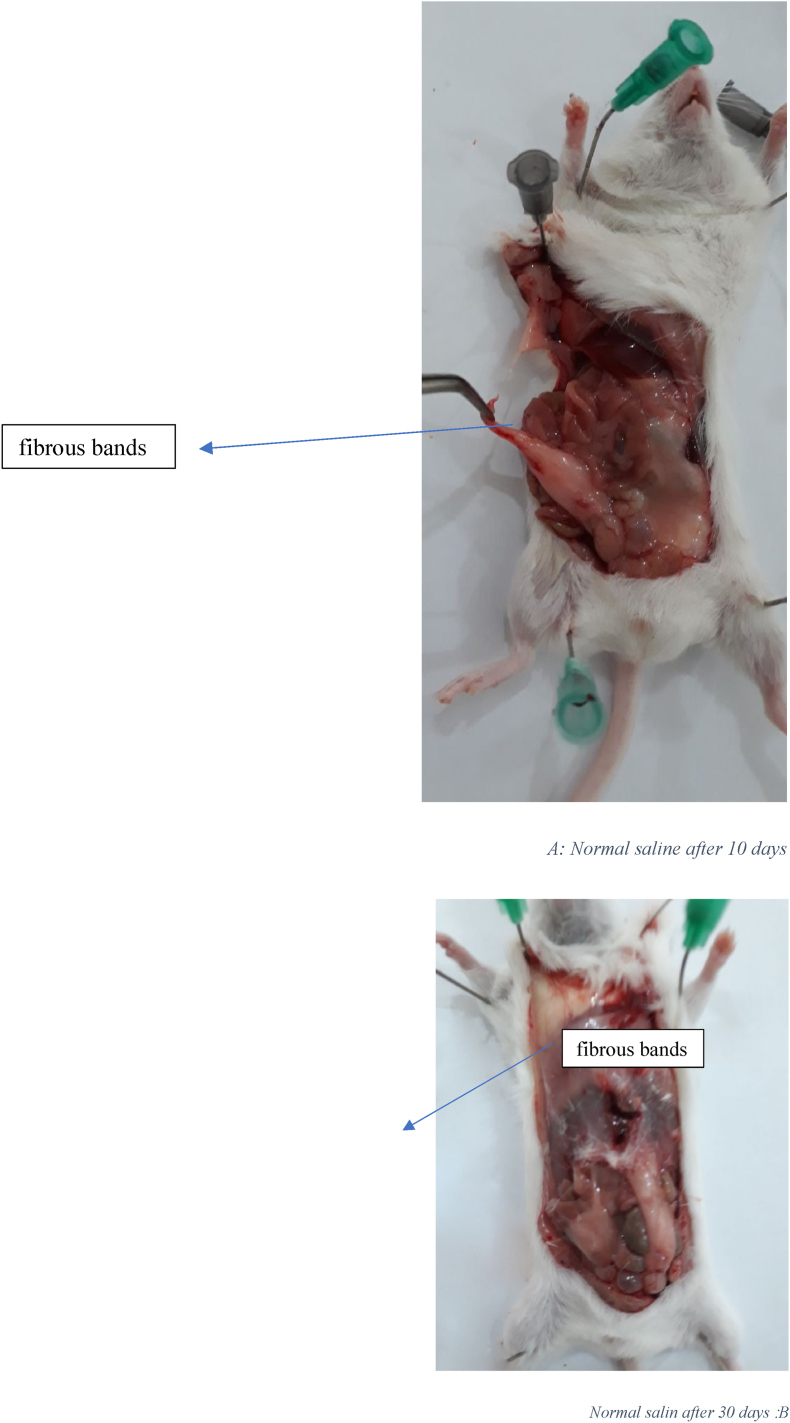

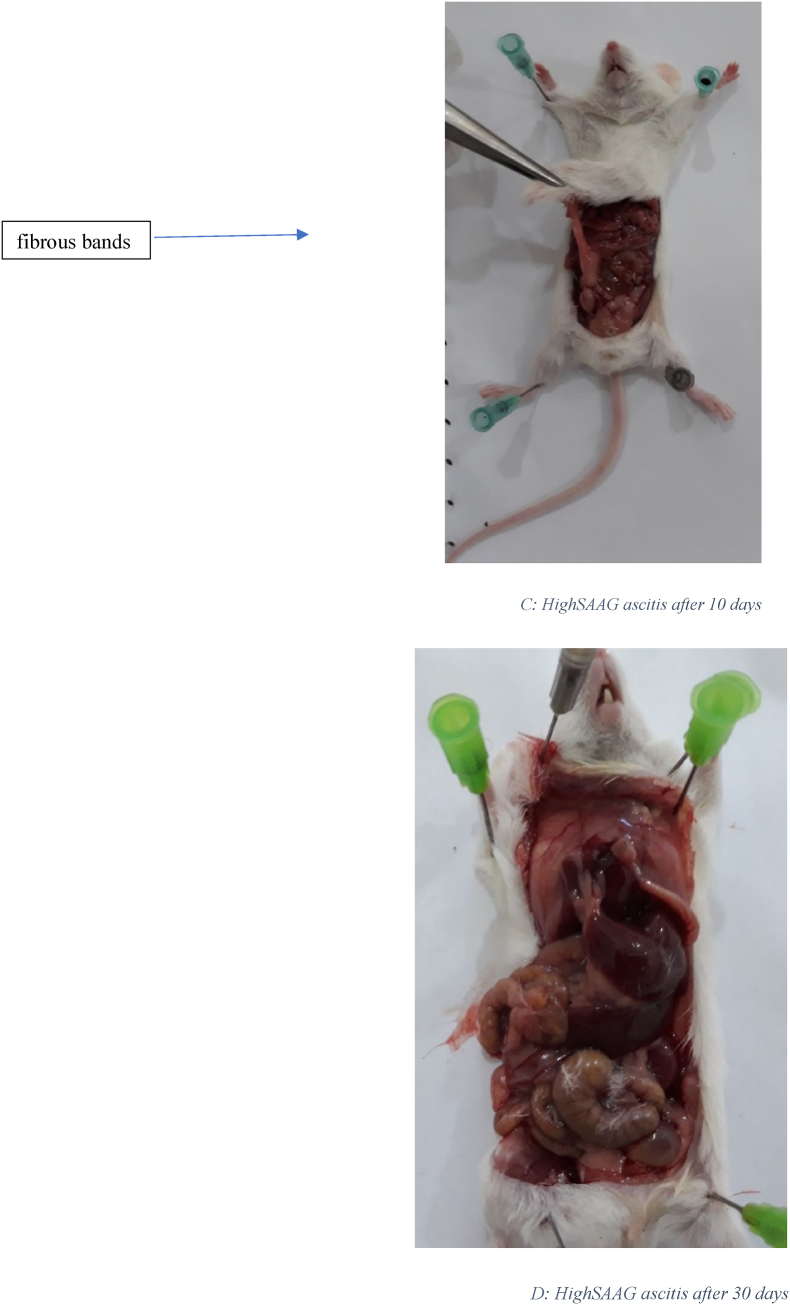

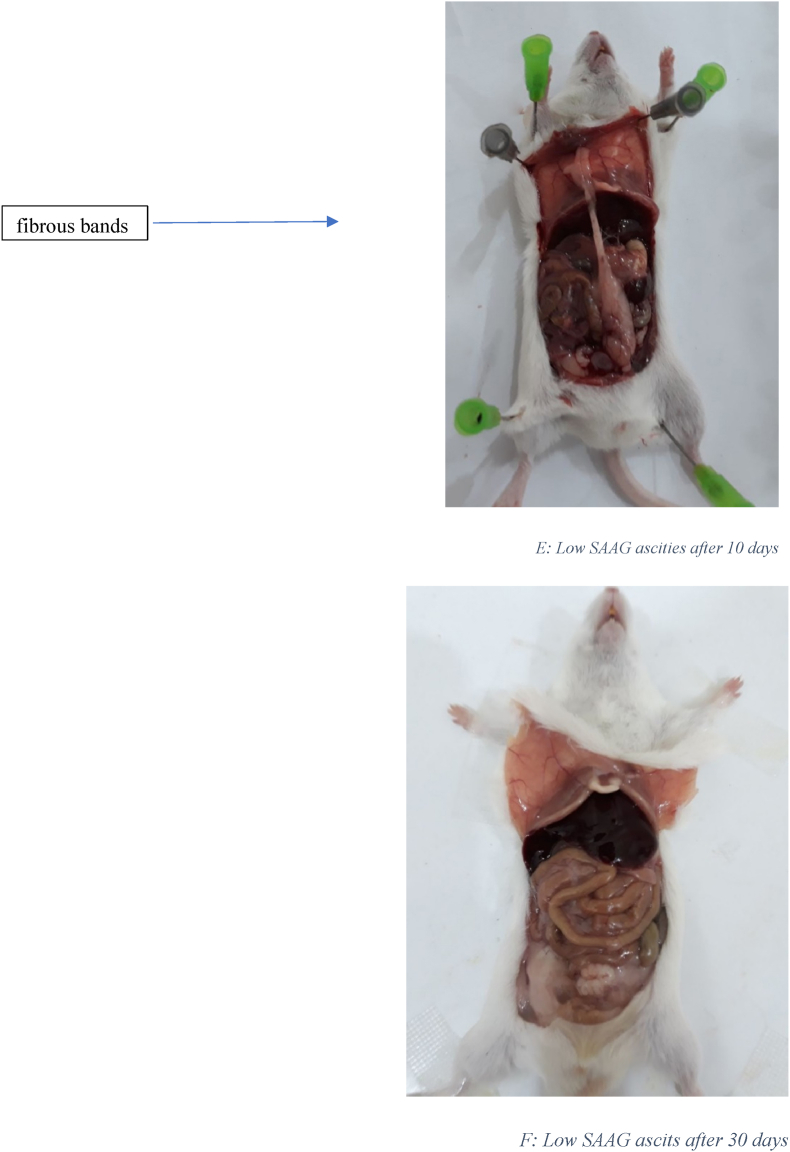
A: Intra-abdominal view10 days after laparotomy in the normal salin group: 8 rats had 3 or more than 3 fibrous bands B: Intra-abdominal view 30 days after laparotomy in the normal salin group: 9 rats had more than 3 fibrous bands C: Intra-abdominal view10 days after laparotomy in High SAAG ascits group: 5 rats had 3 or more than 3 fibrous bands D: Intra-abdominal view 30 days after laparotomy in High SAAG ascits group: 6 rats had 3 or more than 3 fibrous band E: Intra-abdominal view10 days after laparotomy in Low SAAG ascits group: 2 rats had 3 fibrous bands, no more than 3 fibrous bands were seen, some adhesion were seen F: Intra-abdominal view 30 days after laparotomy in Low SAAG ascits group: 2 rats had 3 fibrous bands, no more than 3 fibrous bands were seen, No adhesion.

Fig. 1 displays the histological images of microscopic adhesion at 10 and 30 days after laparotomy in the various research groups. Fig. 2 shows the intra-abdominal images of rats in various research groups 10 and 30 days following laparotomy.

## 4 Discussion

Based on the results of the present study, in the microscopic examination after 10 days and after 30 days, the lowest rate of adhesion was significantly followed by the injection of Low SAAG ascites fluid. Additionally, Low SAAG ascites fluid has been shown to clearly and substantially reduce intra-abdominal adhesions in studies measuring macroscopic adhesions after 10 and 30 days following laparotomy. One possible method of action is that the results of inflammatory processes may be diluted and transmitted to the lymph by being injected with ascites as a physiological fluid. This may lead us to the ideal fluid composition to prevent intraabdominal adhesions.

Intra-abdominal adhesions are secondary to peritoneal injuries such as previous surgery or intra-abdominal infections. Therefore, it is important to use appropriate methods to minimize the possibility of adhesions in any surgery. In our clinical practice we observed that patients with ascites experienced less complications attributable to intraabdominal adhesion, in post laparotomy setting. In this regard, we made the decision to research the impact of induced ascites in the form of High SAAG and Low SAAG on the frequency of microscopic and macroscopic adhesion after surgery in a mouse model due to the absence of comparable studies.

In a study by Aarons et al. They inspected the role of statins in reduction of postoperative adhesion formation. They induced adhesions in rats (n = 102). Afterward, rats received intraperitoneal lovastatin (30 mg/kg), or atorvastatin (30 mg/kg) at the time of laparotomy. After 7 days relaparotomy indicated that Lovastatin and atorvastatin reduced intraabdominal adhesion development by 26% and 58%, respectively (P < 0.05) which was consistent with our study and the fact that the ascites fluid (both High SAAG and low SAAG) used in our study happened to be more efficient in preventing the adhesion band formation compared to the control group. (10). In 2009, Aksoy et al. evaluated three groups of 36 Wistar albino male rats to assess the effectiveness of glycerin-induced fake ascites in preventing abdominal adhesions. When compared to the control and normal saline groups, relaparotomy performed after 10 days demonstrated that the group receiving artificial ascites (glycerin) had the lowest intraabdominal adhesion rate (7). This evidence implies that the appliance of any form of ascites may act as a mechanical barrier among the injured surfaces of the tissues or dissolve the fibrin formation on that area. A study by Soltani et al., In 2005 compared the effect of washing the peritoneal cavity for 3 min with mitomycin and dexamethasone and normal saline solutions on intra-abdominal adhesion in mice after 6 weeks. 60 rats were employed, split into 3 groups. In comparison to dexamethasone and normal saline, mitomycin reduced intra-abdominal adhesion in rats (11). These studies' findings agreed with those of ours. To validate the function of anti-inflammatory drugs in achieving these effects, more research must be conducted. Octavin et al. conducted a retrospective study in Romania in 2018 entitled “Using methylene blue to prevent recurrent intra-abdominal adhesions after surgery” which used methylene blue during surgery of 20 patients presented with bowel obstruction. The mean fallow up duration was 28.5 months. They found that utilization of methylene blue in surgery is associated with reduced adhesion-related symptoms (complete and incomplete obstruction). Methylen blue is believed to enhance peritoneal fibrinolytic activity. (12).

The most comparable studies that have been conducted to date were compared and examined with this investigation, since the examination and assessment of publications and journals of comparable studies to the current study did not provide any findings. One limitation implacable to our study is that we used human derived ascites and we were not able to exactly point out the potential effective ingredient of the ascites fluid in the prevention of adhesion formation. However, the comparison of biochemical analysis of both low and high SAAG ascites fluids, may be a guide for future studies to determine the ultimate effective composition of an artificial ascites fluid. Further studies on this matter are recommended.

## 5 Conclusion

By comparison, the least microscopic and macroscopic IAAs, 10 and 30 days after surgery, were found in the low SAAG ascites group. After 10 days of surgery, macroscopic IAA in the high SAAG group was significantly lower than that in the other control and Low SAAG groups. The main avoidance of adhesion and postoperative problems may directly correlate with lower IAAs utilizing low SAAG ascites fluids. The findings recommend comparing the outcomes in bigger mouse populations or even after creating simulated sterile ascites fluid in human populations. More experiments with bigger populations may lead to more trustworthy findings.

A: The histopathological view of microscopic adhesion 10 days after laparotomy in normal saline group: moderate to severe infiltration of lymphoplasmacells and intercellular edema. Significant amount of fibrosis and connective tissue formation on the serosa B: The histopathological view of microscopic adhesion 10 days after laparotomy in normal saline group: moderate to severe infiltration of lymphoplasmacells and intercellular edema. Significant amount of dens fibrosis and sever connective tissue formation on the serosa C: The histopathological view of microscopic adhesion 10 days after laparotomy in High SAAG ascites group: Serosa with mild to moderate infiltration of lymphoplasmacells and few neutrophils with ectatic small vessels and a few intercellular edema D: The histopathological view of microscopic adhesion 30 days after laparotomy in High SAAG ascites group: Serosa with mild to moderate infiltration of lymphoplasmacells and few neutrophils with ectatic small vessels and a few intercellular edema E: The histopathological view of microscopic adhesion 10 days after laparotomy in Low SAAG ascites group: Serosa with mild infiltration of lymphoplasmacells and few neutrophils with ectatic small vessels and a few intercellular edema F: The histopathological view of microscopic adhesion 30 days after laparotomy in Low SAAG ascites group: Serosa with mild infiltration of lymphoplasmacells with ectatic small vessels and a few intercellular edema.

## Availability of data and materials statement

The datasets used and/or analyzed during the current study are available from the corresponding author on reasonable request.

## Provenance and peer review

Not commissioned, externally peer-reviewed.

## Ethical approval

The animal ethics committee of Birjand University of Medical Sciences (Ref: IR. BUMS.REC.1399.503).

## Sources of funding

No funding source to declare.

## **Author contribution**

Zahra Amirian and Mohammadreza Ghasemian: Study design and calculating, Zahra Amirian & Mohsen Najmodini: Writing and editing, Mahmoud Zardast: Pathology Report.

## Registration of research studies

1. Name of the registry: NO
2. Unique Identifying number or registration ID: NO
3. Hyperlink to your specific registration (must be publicly accessible and will be checked): NO

## Guarantor

Zahra Amirian and Mohammadreza Ghasemian.

## Declaration of competing interest

The authors declare no competing interest.
